# Young investigators in natural products chemistry, biosynthesis, and enzymology

**DOI:** 10.3762/bjoc.20.229

**Published:** 2024-10-29

**Authors:** Jeffrey D Rudolf, Lena Barra, Takayoshi Awakawa

**Affiliations:** 1 Department of Chemistry, University of Florida, PO Box 117200, Gainesville, FL 32611, USA; 2 Fachbereich Chemie, Universität Konstanz, Universitätsstraße 10, 78464 Konstanz, Germany; 3 RIKEN Center for Sustainable Resource Science, Wako, Saitama 351-0198, Japan

There is just something about natural products. When staring at these structurally complex molecules produced by diverse organisms on our planet, one may wonder why a particular natural product is made, how it is made, how I can make it, or how I can use it. The isolation and structural elucidation of a single natural product can inspire a wide array of scientific disciplines. Natural products, first as bioactive metabolites in traditional medicines and now as isolated or (bio)synthesized metabolites, are one of the most important sources of therapeutics historically and today. Their privileged structures have led organic and bioorganic chemists to develop methods to construct them. Our fundamental knowledge in enzymology is continually expanded by enzymes involved in natural products biosynthesis, as their production requires evolved enzymes to perform chemical reactions distinct from those seen in primary metabolism.

Natural products have inspired generations of scientists and continue to do so today. This thematic issue highlights young investigators in natural products chemistry, biosynthesis, and enzymology. As early-career researchers ourselves, we are honored to present this collection of 19 Full Research Papers, three Letters, and nine Review articles from our colleagues around the world. Topics covered include natural product discovery efforts from a variety of organisms, genome and transcriptome mining and bioinformatics of natural products, biosynthetic gene clusters, and enzymes, development of chemical probes, biocatalysis and chemoenzymatic total synthesis, enzymatic mechanisms, and computational investigations of chemical structures and reactions. All of the major classes of natural products are represented here: nonribosomal peptides, ribosomally-synthesized and posttranslationally modified peptides, polyketides, alkaloids, aromatics, and terpenoids. This generation of young investigators will continue to shape the field of natural products for years to come.

We pass on our greatest thanks to all the contributors to this special thematic issue. We also humbly thank Prof. Jeroen Dickschat for inviting each of us to guest-edit this wonderful issue featuring rising stars in the field of natural products. Finally, we thank the staff of the *Beilstein Journal of Organic Chemistry* for making this issue possible. As you browse this issue, we hope that you are inspired, not only by the science but by the young researchers contributing to this field. Keep an eye on these early-career researchers; the future of natural products research is in very capable hands!

Jeff Rudolf, Lena Barra, and Takayoshi Awakawa

Gainesville, Konstanz, Wako, September 2024

## Data Availability

Data sharing is not applicable as no new data was generated or analyzed.

